# Impact of Unmet Care Needs on Resilience in Multimorbid Older Adults

**DOI:** 10.1093/geroni/igaf122.3962

**Published:** 2025-12-31

**Authors:** Boah Kim, Lun Li, Andrew Wister

**Affiliations:** Gerontology Research Centre, Simon Fraser University, Vancouver, British Columbia, Canada; MacEwan University, Edmonton, Alberta, Canada; Simon Fraser University, Vancouver, British Columbia, Canada

## Abstract

Older adults with multimorbidity (i.e., two or more chronic conditions) are more likely to face an enduring risk of unmet (care) needs in accessing healthcare services due to their unique and complex form of health adversity. Multimorbidity resilience (MR), the ability to bounce back from multiple chronic illness-related challenges, is an important indicator of positive adaptation to multimorbidity. However, despite the importance of addressing unmet needs among multimorbid older adults, there is sparse knowledge on the impact of unmet needs on resilience. This study aims to fill these gaps through an investigation of the key factors affecting resilience among multimorbid older adults. A social determinant of health model is used to frame the study. Using the Canadian Longitudinal Study on Aging (CLSA), a total of 12,677 multimorbid older adults were analyzed. Hierarchical linear regression was conducted to identify the relationship between resilience and four sequentially ordered blocks of predictors: 1) unmet needs, 2) sociodemographic characteristics, 3) health context, and 4) social/environmental supports. It was found that the level of resilience was lower among multimorbid older adults who reported having unmet needs compared to those without unmet needs. Several significant covariates across multiple domains were also identified (e.g., sex, urban/rural status, household income, perceived healthy aging, community friendliness). This study extends our understanding of potential protective and/or risk factors of MR among older adults. To address the challenges of accessing healthcare services among multimorbid older adults, there is a need to implement health policies/initiatives to fill their unmet care needs.

